# Exchange Journal

**Published:** 1836

**Authors:** 


### IV.—ORIGINAL.

### The Western Journal.

*E Sylvis Nuncius.*

CINCINNATI, JULY 1836.

### Art. I—Exchange Journals.

We have lately received several new works in exchange for our Journal, from which we expect to extract whatever we may deem interesting to our readers. Among the most valuable we readily place a London quarterly, conducted by Drs. Forbes and Conolly, editors of the Cyclopædia of Practical Medicine, a valuable English publication. We have also received the India Journal of Medical Sciences, the only periodical of the character published in India. It is issued monthly from a Calcutta press, and embraces much that is useful and unique in the profession, at least to us Americans. Its editorial department appears to be under the direction of F. Corbin, Esq., and, judging from the volume sent us, it must contribute much to our knowledge of the diseases of that country. The London Quarterly is entitled "The British and Foreign Review," and is republished in Baltimore under the direction of Prof. Dunglission, whose learning and talents are already known to the profession. It is arranged after the plan of the Medico-Chirurgical Review, edited by Dr. Johnson. We shall take much pleasure in enriching the pages of our periodical from both these valuable works.

